# Enterovirus D68 in Hospitalized Children: Sequence Variation, Viral Loads and Clinical Outcomes

**DOI:** 10.1371/journal.pone.0167111

**Published:** 2016-11-22

**Authors:** Katherine Moyer, Huanyu Wang, Douglas Salamon, Amy Leber, Asuncion Mejias

**Affiliations:** 1 Department of Pediatrics, Division of Infectious Diseases, Nationwide Children’s Hospital, Columbus, Ohio, United States of America; 2 Department of Pathology and Laboratory Medicine, Nationwide Children’s Hospital, Columbus, Ohio, United States of America; 3 Center for Vaccines and Immunity, The Research Institute at Nationwide Children’s Hospital, The Ohio State University, Columbus, Ohio, United States of America; University of North Carolina at Chapel Hill School of Dentistry, UNITED STATES

## Abstract

**Background:**

An outbreak of enterovirus D68 (EV-D68) caused severe respiratory illness in 2014. The disease spectrum of EV-D68 infections in children with underlying medical conditions other than asthma, the role of EV-D68 loads on clinical illness, and the variation of EV-D68 strains within the same institution over time have not been described. We sought to define the association between EV-D68 loads and sequence variation, and the clinical characteristic in hospitalized children at our institution from 2011 to 2014.

**Methods:**

May through November 2014, and August to September 2011 to 2013, a convenience sample of nasopharyngeal specimens from children with rhinovirus (RV)/EV respiratory infections were tested for EV-D68 by RT-PCR. Clinical data were compared between children with RV/EV-non-EV-D68 and EV-D68 infections, and among children with EV-D68 infections categorized as healthy, asthmatics, and chronic medical conditions. EV-D68 loads were analyzed in relation to disease severity parameters and sequence variability characterized over time.

**Results:**

In 2014, 44% (192/438) of samples tested positive for EV-D68 vs. 10% (13/130) in 2011–13 (p<0.0001). PICU admissions (p<0.0001) and non-invasive ventilation (p<0.0001) were more common in children with EV-D68 vs. RV/EV-non-EV-D68 infections. Asthmatic EV-D68+ children, required supplemental oxygen administration (p = 0.03) and PICU admissions (p <0.001) more frequently than healthy children or those with chronic medical conditions; however oxygen duration (p<0.0001), and both PICU and total hospital stay (p<0.01) were greater in children with underlying medical conditions, irrespective of viral burden. By phylogenetic analysis, the 2014 EV-D68 strains clustered into a new sublineage within clade B.

**Conclusions:**

This is one of the largest pediatric cohorts described from the EV-D68 outbreak. Irrespective of viral loads, EV-D68 was associated with high morbidity in children with asthma and co-morbidities. While EV-D68 circulated before 2014, the outbreak isolates clustered differently than those from prior years.

## Introduction

Enterovirus D68 (EV-D68) was originally identified in 1962 in four children with severe respiratory tract infection in California. [1] Since then, the circulation of EV-D68 has been limited to individual cases and small outbreaks as reported by the National Enterovirus Surveillance System (NESS). [2–6] In late summer of 2014, a large outbreak of EV-D68 associated with severe respiratory illness was identified in the Midwestern United States (US). [7] Studies showed that the EV-D68 outbreak strains were associated with respiratory distress and disproportionately affected asthmatic children compared with rhinovirus/enterovirus (RV/EV-non-EV-D68) infections, which are the most common infectious triggers of asthma. [8–14] Those studies, however, were mainly focused in otherwise healthy children and included limited data regarding the disease spectrum of EV-D68 in children with underlying medical conditions other than asthma. [10, 13, 14]

During the outbreak, the sequence variability of the EV-D68 strains from the US was compared with worldwide sequences, and it was shown that these EV-D68 strains mainly belonged to clade B. [15, 16] The characterization and circulation of EV-D68 strains within the same institution over time, and whether EV-D68 genomic loads are associated with enhanced clinical disease has not been described.

The objectives of this study were to define the clinical and virologic impact of EV-D68 in a large cohort of children, including those with chronic medical conditions, hospitalized from May 2014 to November 2014 at our center. We also sought to determine the impact of EV-D68 loads and the sequence variability of EV-D68 isolates identified during the outbreak compared to strains circulating in previous years (2011–2013) in our community.

## Materials and Methods

### Patient Population and Study Design

Nasopharyngeal (NP) swabs from patients <21 years of age admitted to Nationwide Children’s Hospital (NCH) with an acute respiratory illness from May to November 2014, and from August to September of 2011, 2012 and 2013 that tested positive for rhinovirus/enterovirus (RV/EV) as part of a PCR panel were identified. From 2011 to 2013 RV/EV identification was performed using a laboratory developed real time (RT)-PCR assay [17], while in 2014 RV/EV were identified using a multiplex PCR panel (FilmArray Respiratory Panel; BioFire, Salt Lake City, UT). Viral testing was performed at the discretion of the attending physician. Nationwide Children's Hospital is the only tertiary care children’s hospital in Central Ohio with 508 beds, 18,160 admissions per year and over 218,596 emergency visits per year.

A convenience sample of specimens that tested positive for RV/EV by either method were selected and subsequently tested for EV-D68 using a laboratory developed single step RT-PCR assay. Samples were selected randomly based on availability, integrity and amount of remnant specimen. EV-D68 testing was completed after the patient encounter, thus results were not available to the physician treating the patient.

Electronic health records (EHR) from all children identified (RV/EV-non-EV-D68 and EV-D68) were reviewed for pertinent demographics, clinical characteristics and outcomes of care including, admission to the pediatric intensive care unit (PICU) and length of PICU stay, administration and duration of supplemental oxygen, need for invasive and non-invasive ventilatory support (specifically continuous positive airway pressure [CPAP] and bilevel positive airway pressure [BiPAP]), and duration of hospitalization. These parameters were compared first among children with RV/EV-non-EV-D68 vs. EV-D68 infections, and then within the EV-D68 group between previously healthy children, children with history of asthma/wheezing and those with chronic medical conditions. The history of asthma/wheezing and chronic medical conditions were first identified by electronic extraction from patients’ EHR and then verified by manual review for accuracy. Patients with no other comorbidities except for history of asthma or any prior wheezing episodes were included in the asthma/wheezing group.

In children with EV-D68 infection, relative viral burden (cycle threshold (Ct) values) was compared and correlated with disease severity parameters. Last, based on availability, we determined in a subset of samples, the local prevalence and differences in sequence homology of EV-D68 strains identified during the 2014 outbreak and prior years (August to September of 2011, 2012 and 2013). The study was approved by the Institutional Review Board of Nationwide Children’s Hospital (IRB#13–00796). Due the retrospective nature of this study, the study involved no more than minimal risk to subjects and a waiver of consent was requested and approved by the IRB.

### EVD68 Identification, quantitation and Sequencing and Analysis

A single step, EV-D68-specific reverse transcriptase real-time PCR was developed in house during the 2014 outbreak. The primer and probe sequences were designed based on *in silico* analysis of all the available EV-D68 sequences deposited in NCBI, targeting the 5’ non-translated region of the human enterovirus genome. The NCBI database was accessed on September 15, 2014 and at that time < 20% of the EVD68 2014 outbreak sequences were included. Briefly, total nucleic acid was obtained by extraction using the NucliSENS easyMag platform (bioMerieux, Durham, NC) and 4μL of the eluate was added to a 20 μL total volume reaction mixture [1XTaqMan RT-PCR mix (TaqMan RNA-to-C_T_ 1-Step Kit, Life Technologies, Grand Island, NY), 1XTaqMan RT Enzyme Mix, 0.25μM of each primer: EVD68F (5’-AAAACCATGACGCTAGACATGA-3’) and EVD68R (5’-GGCCGGAGGACTCTAT-3’) and 0.9μM of probe EVD68P (5’-VIC-CAAGGTGTGAAGAGTCTATT- MGB-3’)]. The RT-PCR was carried out using the ABI 7500 thermocycler (Life Technologies, Grand Island, NY) with the following running conditions: 48°C for 15 min, followed by 10 min at 95°C, 40 cycles of 95°C for 15s and 50°C for 1 min. Semiquatitative genomic EV-D68 loads were quantified using Ct values, which reflect the number of amplification cycles that are required for a positive PCR test. Thus the higher the Ct value the lower the amount of genomic EV-D68.

For EV-D68 sequencing, partial VP1 genes from EV-D68 strains identified at our hospital during the 2014 outbreak and in previous years, were amplified to yield an 805-basepair (bp) PCR product, as described. [18] Cycle sequencing was performed with BigDye Terminator v3.1 cycle sequencing Kit (Applied Biosystems) on the automated sequencer 3130xl Genetic Analyzer (Applied Biosystems) bi-directionally. Multiple sequence alignment was performed and phylogenetic trees were generated using the neighbor-joining method implemented in MEGA version 6.0. [19] Support for specific tree topologies was estimated by bootstrap analysis with a value of 1000. Using Geneious software (http://www.geneious.com,[20]), the amino acid sequences (including BE and DC loops) of partial VP1 were aligned and compared with NCH strains and selected sequences that were previously published.

### Statistical Analysis

Descriptive analyses were performed using frequency distributions or rates. Medians (percentiles 25th-75th) were used to summarize the demographic data and patient’s baseline characteristics. Associations between categorical and continuous variables were analyzed using the Fisher’s exact tests, Chi-square, and the two-tailed Student’s T tests or Mann-Whitney test where appropriate. Normality for continuous variables was checked using Shapiro-Wilk test. The Bonferroni correction was applied to correct for multiple testing when analyzing families that included multiple parameters such as signs/symptoms at presentation or medications used. Statistical analyses were performed using GraphPad Prism 6.

## Results

### Patient Population

**Fig 1:**
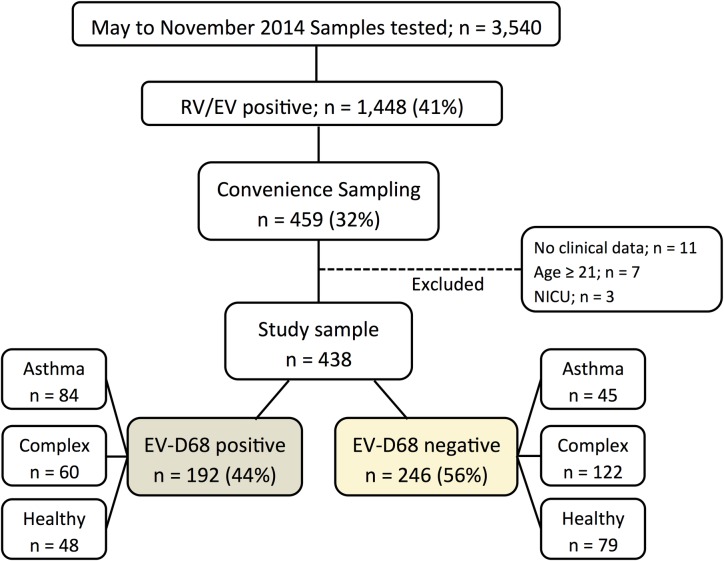
Sample and patient selection for the EV-D68 outbreak in 2014. From May to November 2014 3,540 samples underwent viral testing at NCH Department of Pathology. Of those 41% tested positive for rhinovirus/enterovirus (RV/EV) by a single or multiplex PCR assay. A convenience sample of all RV/EV positive specimens was selected randomly based on availability, integrity and amount of specimen. Of this convenience sampling 44% were positive for EV-D68.

From May 19 to November 26, 2014, 3,540 nasopharyngeal samples from children with respiratory symptoms underwent viral testing at Nationwide Children’s Hospital Department of Microbiology. Of those, 1,448 (41%) samples tested positive for RV/EV. After excluding duplicate samples from the same or subsequent admissions, 32% (459/1448) of the RV/EV positive samples were randomly selected based on availability, integrity and amount of remnant specimen. These 459 samples were then tested for EV-D68 using a laboratory developed RT-PCR assay (Fig 1). We excluded from the analyses samples from patients in whom clinical data was not available, patients ≥ 21 years of age, and infants hospitalized in the neonatal intensive care unit (NICU; n = 4). The four NICU patients were excluded because they had been hospitalized already for a prolonged period of time when they tested positive for EV-D68, and had additional risk factors for oxygen requirement and extensive length of stay. Of the remaining 438 RV/EV positive samples, 192 (44%) tested positive for EV-D68.

**Fig 2:**
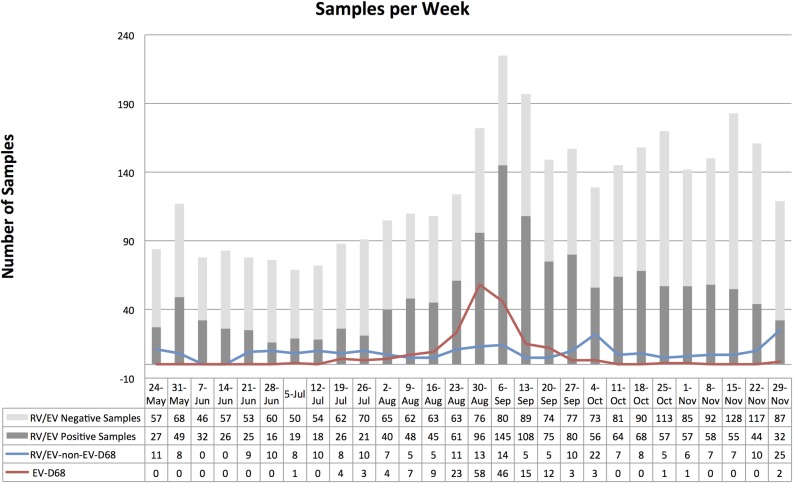
Number of samples tested for respiratory viruses per week in 2014. The y-axis represents the number of samples per week. The x-axis represents the number of samples that underwent viral testing and those positive for rhinovirus/enterovirus per week from May 24 to November 29 2014. The stacked bars represent total number of respiratory samples tested for any respiratory virus. The light gray section of the bar represents the number of samples that tested negative for RV/EV while the dark gray section indicate the number of samples positive for RV/EV. The colored lines represent the convenience sampling, in blue the RV/EV-non-EV-D68 positive samples and in red the positive EV-D68 samples.

During the time period specified, the total number of samples sent for testing, along with those that tested positive for RV/EV and EV-D68 are depicted in Fig 2. The peak of EV-D68 positivity was identified from 8/23/14 to 9/20/14, while RV/EV detection was constant throughout the study period.

### Clinical Characteristics and Outcomes of Children with Rhinovirus/Enterovirus-non EV-D68 and EV-D68 Infections during the 2014 Outbreak

We compared the clinical characteristics and severity of patients that tested positive for EV-D68 versus those that tested positive for RV/EV but negative for EV-D68 (RV/EV-non-EV-D68) during the outbreak (Table 1). Children with EV-D68 infection were significantly older, with no overall differences in sex or race. Body mass index (BMI) was within normal range for both groups, although it was significantly higher in children with EV-D68 infection. History of asthma was as well more common in EV-D68 positive children, while underlying chronic medical conditions were more commonly present in RV/EV-non-EV-D68 children, and included genetic syndromes, congenital heart disease, history of prematurity, primary or acquired immunodeficiency, neurologic disorders and neuromuscular disorders.

**Table 1:** Demographics and Clinical Characteristics of children with EV-D68 or Non-EV-D68 RV/EV infection during the 2014 Outbreak.

		EVD68 Positive n = 192	Non-EV-D68 RV/EV n = 246	p-value
**Demographic Information**				
Age (years)		5.0 (2.0–7.7)	1.9 (0.4–5.1)	<0.0001
Sex, male, n (%)		112 (58.3)	139 (56.5)	0.77
Race/ethnicity				
	White	79 (41.1)	132 (53.7)	0.08
	African American^(1)^	75 (39.1)	71 (28.9)	
	Biracial	22 (11.5)	28 (11.4)	
	Hispanic	10 (5.2)	11 (4.5)	
	Other^(2)^	6 (3.1)	4 (1.6)	
BMI		17.0 (15.5–19.2)	16.4 (15.0–18.4)	0.006
**Underlying medical conditions**				
	Healthy	48 (25.0)	79 (32.1)	<0.0001
	Chronic medical conditions^(3)^	60 (31.3)	122 (49.6)	
	Asthma	84 (43.8)	45 (18.3)	
**Symptoms/Signs, n (%)**				
	Maximum temperature (°C)	38 (37.7–38.3)	38 (37.4–38.7)	0.92
	Respiratory distress	150 (78.1)	121 (49.2)	<0.001
	URI symptoms	168 (87.5)	205 (83.3)	0.28
	Wheezing	142 (74.0)	74 (30.0)	<0.001
	Hypoxia	44 (22.9)	45 (18.3)	0.23
	Vomiting	24 (12.5)	43 (17.5)	0.18
	Rash	10 (5.2)	19 (7.7)	0.33
	Seizure	4 (2.1)	11 (4.5)	0.19
**Medications**				
	Systemic steroid	168 (87.5)	115 (46.7)	<0.0001
	Bronchodilator	175 (91.1)	133 (54.1)	<0.0001
	Inhaled steroid	121 (63.0)	81 (32.9)	<0.0001
	Magnesium	121 (63.0)	63 (25.6)	<0.0001
**CXR findings**				
	Normal	26 (17.8)	32 (22.9)	0.66
	BWT/hyperinflation	51 (34.9)	40 (28.6)	
	Interstitial markings/atelectasis	44 (30.1)	48 (34.3)	
	Lobar consolidation	12 (8.2)	8 (5.7)	
	Pleural effusion	5 (3.4)	3 (2.1)	
	Other	9 (6.2)	9 (6.4)	
**CBC with differential**^(4)^				
	WBC/mm^3^	11,850 (8,400–15,800)	11,200 (7,500–15,100)	0.28
	Lymph %	14 (8–26)	25 (13–42.5)	<0.0001
	Neutrophil %	71 (55.5–79)	57(32.5–96)	<0.0001
	Monocytes %	6 (10.3–21)	7 (4–12)	0.07
	Eosinophil %	0.5 (0–3)	0 (0–2)	0.50

Continuous variables reported in medians, 25–75% interquartile ranges unless otherwise specified. The Bonferroni correction was applied to correct for multiple testing when analyzing families that included different parameters such as signs/symptoms at presentation or medications used.

**(1)** Post-hoc analyses identify a significantly higher proportion of African American children with EV-D68 infection (p = 0.03)

**(2)** Other includes Asian, Native Hawaiian and unknown.

**(3)** Chronic medical conditions included genetic syndromes, congenital heart disease, prematurity, primary or acquired immunodeficiency, neurologic disorders including seizures, developmental delay, and neuromuscular disorders.

CXR: chest-x-ray; BWT: bronchial wall thickening; CBC: complete blood count.

**(4)** CBC with differential was obtained in 82 patients with EV-D68 and 133 patients with non-EV-D68.

Regarding the signs and symptoms at presentation, fever was not high and median temperature was comparable between groups (38°C). Patients with EV-D68 presented more frequently with respiratory distress and with wheezing as per provider documentation. Accordingly, use of systemic or inhaled steroids, bronchodilators, and intravenous magnesium was more common in these children. Chest x-rays were more frequently obtained in children with EV-D68 vs. RV/EV-non-EV-D68 infections (76% vs. 57% respectively; p<0.001) and the most common radiologic finding in both groups was bronchial wall thickening, interstitial markings and atelectasis. Lobar consolidation was uncommon and identified in 6% and 8% of RV/EV-non-EV-D68 and EV-D68 children respectively.

White blood cell and eosinophil counts were comparable in both groups, however neutrophil percentages were higher in children with EV-D68 infection. Blood and urine cultures were obtained in 41% and 25% of patients with RV/EV-non-EV-D68 respectively, compared with 0.5% and 11% of patients with EV-D68 infection, respectively (p<0.001). The only positive blood culture in the RV/EV-non-EV-D68 group revealed *Streptococcus pneumoniae*. Three children with EV-D68 and one with RV/EV-non-EV-D68 had a concomitant urinary tract infection. The following uropathogens were identified *Enterobacter cloacae*, *Escherichia coli*, *Morganella morganii* and *Proteus mirabilis*. Co-detection with other respiratory viruses was less common in children that tested positive for EV-D68 (2% vs. 8% in RV/EV-non-EV-D68 patients; p<0.01). Of the four children with EV-D68 infection that had another respiratory virus identified, parainfluenza virus (PIV type 2 and 3) was detected in two children, adenovirus in one and influenza A in another child. In 20 children with RV/EV-non-EV-D68 infections another respiratory virus was identified and included: PIV 2, 3 and 4 (n = 7), RSV (n = 5), adenovirus (n = 3), coronavirus (n = 2), influenza A (n = 1), human metapneumovirus (n = 1) and both *Mycoplasma pneumoniae* and RSV in one patient.

Clinical outcomes were compared between children with EV-D68 vs. RV/EV-non-EV-D68 identification (Table 2). Children with EV-D68 were more frequently treated in the PICU and stayed significantly longer in the hospital, with no differences in PICU length of stay compared with RV/EV-non-EV-D68 positive children. In addition, supplemental oxygen and non-invasive ventilatory support were more frequently administered to children with EV-D68 infection compared to those with RV/EV-non-EV-D68, however there was no difference in duration of supplemental oxygenation. Both hospitalization charges and pharmacy charges were higher for children with EV-D68 infection.

**Table 2:** Clinical Outcomes of Children with EV-D68 or RV/EV infections during the 2014 Outbreak.

		EVD68 n = 192	Non-EV-D68 RV/EV n = 246	p-value
**Admission Information**				
	PICU, n (%)	131 (68.2)	95 (38.6)	<0.0001
	PICU LOS (h)	42.5 (26.2–70.5)	46.4 (28.6–117)	0.18
	Hospital LOS (h)	68.0 (45.1–99.7)	54.3 (29.9–96.4)	<0.001
**Ventilation Information**				
	Oxygen administered, n (%)	173 (90.1)	139 (56.5)	<0.0001
	Duration of oxygen (h)	21.7 (6.3–41.7)	20.4 (4.7–54.5)	0.91
	Non-invasive ventilation, n (%)	90 (46.9)	44 (17.9)	<0.0001
	Invasive ventilation, n (%)	8 (4.2)	22 (8.9)	0.06
**Hospital and Pharmacy Charges**				
Hospitalization Charges		$26,724 ($13,683-$44,771)	$17,601 ($9,028-$36,819)	0.0001
Pharmacy Charges		$2,489 ($1,237-$4,660)	$1,183 ($183-$3,946)	<0.0001

Continuous variables reported in medians, 25–75% interquartile ranges unless otherwise specified. PICU: pediatric intensive care unit; LOS: length of stay; h: hours

### Burden of EV-D68 in children with chronic medical conditions

Children with EV-D68 infections were further classified based on past medical history into three groups: previously healthy (n = 48); history of asthma or wheezing (n = 84); and chronic medical conditions other than asthma (n = 60), and their clinical presentation and disease severity parameters compared (Table 3). Children with complex medical conditions included those with genetic syndromes, congenital heart disease, history of prematurity, primary or acquired immunodeficiency, sickle cell disease, neuromuscular and neurologic disorders.

**Table 3:** Demographics, Clinical Characteristics, and Clinical Outcomes of Children with EV-D68 Infection.

		Previously Healthy (n = 48)	Chronic Medical Conditions ^(1)^ (n = 60)	History of Asthma/Wheezing (n = 84)	p-value
**Demographics**					
	Age (y)	4.3 (1.3–6.9)	4.75 (1.6–7.8)	5.9 (3.4–8.7)	0.02
	Sex male, n (%)	30 (62.5)	34 (56.7)	48 (57.1)	0.79
	Race/Ethnicity, n (%)				
	White	30 (62.5)	24 (40.0)	25 (29.8)	0.002
	African American	9 (18.8)	20 (33.3)	46 (54.8)	
	Biracial	6 (12.5)	8 (13.3)	8 (9.5)	
	Hispanic	2 (4.2)	6 (10.0)	2 (2.4)	
	Other^(2)^	1 (2.1)	2 (3.3)	3 (3.6)	
**Symptoms/Signs, n (%)**					
	Maximum Temperature (°C)	38.2 (37.7–38.6)	38 (37.7–38.4)	37.8 (37.7–38.1)	0.001
	Respiratory distress	34 (70.8)	43 (71.7)	73 (86.9)	0.21
	URI symptoms	43 (89.6)	51 (85.0)	74 (88.1)	0.75
	Wheezing	32 (66.7)	40 (66.7)	70 (83.3)	0.21
	Hypoxia	9 (18.8)	19 (31.7)	16 (19.0)	0.15
	Vomiting	7 (14.6)	9 (15.0)	8 (9.5)	0.54
	Rash	3 (6.3)	4 (6.7)	3 (3.6)	0.66
	Seizures	1 (2.1)	3 (5.0)	0 (0)	0.12
**Clinical Outcomes**					
	PICU, n (%)	23 (47.9)	40 (66.7)	68 (81.0)	0.0004
	LOS PICU (hours)	26.4 (19.6–50.2)	60.0 (34.2–122.2)	40.6 (26.5–60.2)	0.004
	O2 required, n (%)	41 (85.4)	51 (85.0)	81 (96.4)	0.03
	Non-invasive ventilation, n (%)	18 (37.5)	29 (48.3)	43 (51.2)	0.30
	Invasive ventilation, n (%)	0 (0)	7 (11.7)	1 (1.2)	0.002
	Duration of O2(hours)	8.2 (3–26.7)	32.6 (13.7–94)	19.5 (6.4–35.8)	<0.0001
	Length of hospitalization (h)	49.7 (39.3–69.5)	90.2 (59.6–185.5)	68.9 (45.6–95.7)	<0.0001
**EV-D68 Ct values**		24.6 (21.6–28.1)	24.6 (21.1–29.9)	26.8 (23.3–30.3)	0.09

Continues variables reported in medians, 25–75% interquartile ranges unless otherwise specified.

(2)Other includes Asian, Native Hawaiian and unknown.

(1) Chronic medical conditions include genetic syndromes, congenital heart disease, prematurity, primary or acquired immunodeficiency, sickle cell disease, and neurologic disorders including seizure, and neuromuscular disorders. URI: upper respiratory infection; PICU: pediatric intensive care unit; O2: oxygen; LOS: length of stay. The Bonferroni correction was applied to correct for multiple testing when analyzing symptoms/signs and clinical outcomes.

Children with history of asthma/wheezing were older than the other two groups and more commonly African American, while the majority of White children were otherwise healthy. Temperature was higher in previously healthy children, with no differences in other signs or symptoms at presentation. Blood cultures were more commonly obtained in patients with chronic medical conditions (47%) compared with otherwise healthy children (2%) or children with asthma (19%; p<0001) and were all negative. Similarly urine cultures were obtained in 22% of children with chronic medical conditions compared to 16% in healthy children and 1% in the asthma group, p<0.001). The three positive urine cultures were all identified in patients with chronic medical conditions who also had genitourinary abnormalities. In regards to clinical outcomes, PICU admission was more common in children with asthma; however, those with complex medical conditions had a significantly longer PICU stay as well as total hospital stay. In addition, patients with chronic medical conditions were more likely to require invasive ventilatory support and received supplemental oxygen significantly longer. No cases of acute flaccid myelitis or deaths were identified in any of the EV-D68 groups.

Relative viral burden (Ct values) was measured in all of the EV-D68 patients and was not significantly different between groups. There were no significant correlations between viral burden and age, length of stay, maximum temperature or duration of supplemental oxygen (p > 0.05). We also compare EV-D68 Ct values between patients requiring PICU admission versus those who were hospitalized in the ward and did not find significant differences (p = 0.90).

### EV-D68 identification in non-outbreak years

Following the scheme used for sample selection during the 2014 outbreak, we randomly selected available NP samples that tested positive for RV/EV from previous years (8/12 through 9/15 of 2011, 2012, and 2013) based on availability and sample integrity. Of the 1,753 samples sent for viral testing during the periods specified, 40% (702/1,753) tested positive for RV/EV. Approximately 20% of positive RV/EV samples (130/702) equally distributed between years (43 in 2011, 43 in 2012 and 44 samples in 2013) were tested for EV-D68 by PCR. Ninety percent of these samples tested negative for EV-D68, while 13 samples (five from 2011, three from 2012, and five from 2013) (10%) tested positive. The proportion of patients with EV-D68 infection from 2011 to 2013 were significantly lower compare with the 2014 outbreak (p <0.001). These patients had a similar presentation to patients diagnosed with EV-D68 during the 2014 outbreak (S1 Fig).

### EV-D68 sequencing across seasons

Based on sample availability and nucleic acid quality, 77 (38%) EV-D68 positive samples (6 from 2011–2013 and 71 from 2014 from Nationwide Children’s Hospital [NCH]) were further sequenced based on the partial VP1 region. Forty-four of the 77 NCH samples (39 from 2014, one from 2012 and four from 2011) had high quality sequences representing the partial VP1 region (corresponding to the 120–750 amino acids position of the prototype Fermon strain), and were included in the analysis. All the NCH strains demonstrated more than 85% sequence identity to the VP1 region of the prototype Fermon strain indicating confirmed identity as EV-D68. [15] The strains were 97.20% identical to each other. Sequence identity was 98.20% among the 39 NCH strains sequenced during the 2014 outbreak and 99.34% among the four 2011 NCH strains. The 2012 strain showed only 88.41% sequence identity to other NCH strains.

**Fig 3:**
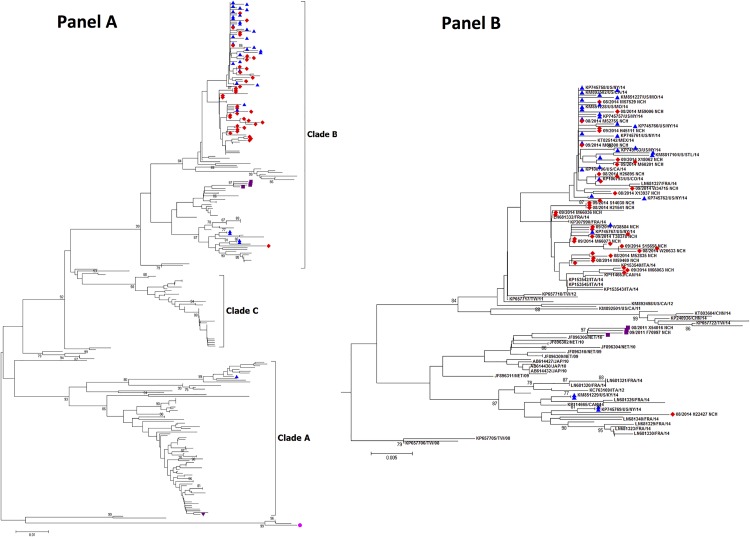
Neighbor-joining phylogenetic tree of EV-D68 strains. **(A)** Phylogenetic tree was constructed using partial viral protein 1 gene sequences of the type strain, Fermon strain (pink circle). Sequences of EVD68 strains from the 2014 outbreak in the US and other geographic areas are depicted in blue triangles and, while sequences from Nationwide Children’s Hospital (NCH) are depicted in red diamonds (2014), purple triangles (2012) and purple squares (2011). Bootstrap values > 75% are shown. The scale bar represents genetic changes in base substitutions per site. (**B)** Subtree of clade B. the legend adjacent to each node reflects the accession number, the location and the year of isolation for each isolate.

Phylogenetic followed by bootstrap analyses indicated that all, but one of the NCH 2014 strains, clustered into a sublineage within the major clade B [4] (Fig 3A & 3B), along with other 2014 US strains [21], suggesting the possibility of a new sublineage. The remaining 2014 NCH strain clustered within clade B but in a different group. Four sequences from 2011 also clustered in clade B along with strains isolated in the Netherlands, but also in a different group. The strain detected in 2012 was found in clade A.

Amino acid substitutions have been related with differences in virulence. To determine if amino acid compositions of the 2011, 2012 and 2014 NCH strains were aligned with other strains in the cluster we performed multi-alignment analysis using Geneious software (S2 Fig). The amino acid composition of the 2014 NCH strains were closely related with viruses that were identified in the US in 2014 with highly similar BC and DE loops. The 2012 NCH strain was closely related with strains isolated from Italy in 2012 [22], displaying four unique substitutions (S95E, I131V, N140S, M148T) and one glycine amino acid deletion at position 141. The four 2011 NCH strains demonstrated similar amino acids signatures compared with the Netherlands strains [23], characterized by the D152N amino acid sequences.

## Discussion

Our study represents one of the largest cohorts of pediatric patients with EV-D68 infection during the 2014 outbreak in the US describing their clinical characteristics, assessment of viral burden and EV-D68 sequence variability over time. We found that EV-D68 caused severe respiratory distress in children during the 2014 outbreak and was associated with remarkable disease severity with over half of patients requiring PICU admission and invasive or non-invasive ventilation. We also found that although severe disease was most prominent in children with a history of asthma or wheezing, EV-D68 had a significant impact on children with other co-morbidities, independent of viral burden. Last, we confirmed that the 2014 EV-D68 strains clustered in clade B, along with other reported US strains, but clustered differently than isolates from previous years, suggesting that the 2014 strains belong to a new sublineage.

Prior studies suggested that EV-D68 infections were more common in older pediatric patients (4–7 years) that presented with severe respiratory distress, affecting patients with a history of asthma disproportionately. [11, 13, 14] In agreement with those studies, we also found that children with EV-D68 presented with severe respiratory distress, regardless of the presence of underlying medical conditions. We also found that African American children were more commonly infected with EV-D68, which has not been reported before and may be related to the higher prevalence of asthma in this population. [24] In addition and contrary to RV/EV-non-EV-D68 infections, co-detection of other viruses or bacteria was uncommon. Although the lack of co-detection with other respiratory viruses could have been related to the seasonality of the outbreak, a time of the year when the circulation of other respiratory viruses is usually low, it also suggests the major role of EV-D68 in association with severe acute respiratory distress.

Contrary to other reports, hospital and pharmacy charges were significantly higher in patients with EV-D68, which may be related to the high proportion of PICU admissions in our patient population. [13] In our study 68% of children with EV-D68 were admitted to the PICU, which is similar to the rates reported by Midgley et al [11], but significantly higher compared to the 20% reported by others. [10, 13, 14] Nevertheless, once admitted, PICU length of stay was comparable between children with EV-D68 vs. those in the RV/EV-non-EV-D68 group. The variance in PICU admissions between our study and others could be attributed to differences in clinical practices followed at different institutions. In our institution, administration of invasive or non-invasive ventilator support, as well as (IV) magnesium, which is associated with hypotension, is managed in the PICU. In the present study, of the 131 children with EV-D68 infection treated in the PICU, 95% received either invasive or non-invasive ventilation and/or IV magnesium, suggesting that EV-D68 patients met appropriate criteria for PICU admission at our institution. No cases of acute flaccid myelitis or deaths, which have been reported in approximately 1% of infected patients, were found in our study. [7, 12]

The burden of EV-D68 in children with chronic medical conditions has not been well characterized. A recent study conducted in the US showed that non-EV-D68 RV/EV infections were more common in children with underlying medical conditions while EV-D68 was identified in about 1/3 of these patients, which is in agreement with our findings. [13] We also found that the presentation of EV-D68 in children with chronic medical conditions was similar to those with asthma or previously healthy, with low grade fever and severe respiratory distress, which was present on admission in 70% to 87% of cases. However, duration of supplemental oxygen, PICU length of stay, and overall duration of hospitalization was significantly longer in those patients compared to previously healthy or asthmatic children. Viral burden in these children was also comparable and did not correlate with clinical outcomes. Thus, while children with a history of asthma or wheezing were more likely to require PICU treatment, children with chronic medical conditions were also affected and carried significant morbidity.

Our findings regarding the detection of EV-D68 in previous years revealed the circulation of EV-D68 before the 2014 outbreak. Phylogenetic and sequence analysis of the partial VP1 region clearly showed that the 2014 NCH strains grouped with other US strains circulating during the 2014 outbreak, in one distinct phylogenetic cluster in clade B, but separately than strains from 2011 that clustered in a different sublineage in clade B, or the 2012 NCH strain that clustered in clade A. Similar to other publications, our findings suggest that these 2014 NCH strains may represent viral evolution and a possible new sublineage [15, 16]. Nevertheless, and although limited because of the small number of patients, the phenotype of children with EV-D68 infection from 2011 to 2013 and 2014 was similar. These findings are in agreement with others who have reported sporadic detection on EV-D68 prior to 2014 with a similar clinical picture. [3, 22, 23]

The analysis of the amino acid sequence focused in the BC and DE loops of the VP1 protein, showed that NCH 2014 strains had a similar pattern of substitutions compared to other 2014 US strains but different compared with strains from prior years. [25] It is known that the VP1 protein is exposed on the surface of the intact virion and contains a serotype specific neutralization site. Nevertheless, the association of these changes and whether they impact neutralization, the host response or the severity of illness are not known. [15]

There are limitations to our study, some of them inherent to a retrospective study design. It is possible that unmeasured confounders and selection bias may have influenced the results since we only analyzed hospitalized patients, representing the more severe forms of the disease. The same type of bias applied to RV/EV-non-EV-D68 patients, which made the groups comparable. Additionally, we used a convenience sampling since not all respiratory samples were available for EV-D68 PCR testing, and thus our cohort may not be a complete representation of all patients that developed EV-D68 infection. Nevertheless, the proportion of samples that tested positive for RV/EV-non-EV-D68 during 2014 and in prior years was 40%, yet the number of cases positive for EV-D68 was significantly higher in 2014. Last, while we were able to compare children with chronic medical conditions with those that were previously healthy or had a history of asthma, we were not able to compare specific conditions such as immunodeficiency or congenital or cardiovascular conditions due to the relatively small number of patients.

In conclusion, in this study due to the large number of pediatric patients with EV-D68 and RV/EV-non-EV-D68 infections identified, we were able to offer a comprehensive clinical and molecular description of EV-D68 infection during the 2014 outbreak. In addition, we identified EV-D68 from previous years and provided both clinical and molecular data for these isolates compared to the outbreak isolates. While a history of asthma has been demonstrated as a potential risk factor for severe disease, EV-D68 may also have a significant impact on children with other co-morbidities. These findings warrant further study and the possible implementation of routine, rapid EV-D68 diagnosis to further define the burden and circulation of EV-D68 in children.

## Supporting Information

S1 Fig Sample selection 2011–2013. From August 12^th^ to September 15^th^ in 2011, 2012 and 2013 samples were selected randomly based on availability, integrity and amount of specimen. Samples included in the analyses were linked with patient characteristics.
(TIFF)

S2 Fig Amino acid signatures identified in the VP1 regions of NCH strains. Partial VP1 protein sequences of NCH strains and selected strains from other areas were compared with the Fermon strain. Colored squares indicate the aminoacid differences compared to the Fermon strain while dots indicate amino acids identical to those in the Fermon strain. The BC loop and the DE loop are included in the black box.
(TIFF)
