# Supplementary figures and images for: Enterovirus D68 in Hospitalized Children: Sequence Variation, Viral Loads and Clinical Outcomes
Source: PLoS One. 2016 Nov 22;11(11):e0167111. doi: 10.1371/journal.pone.0167111 (PMC5119825; doi:10.1371/journal.pone.0167111)

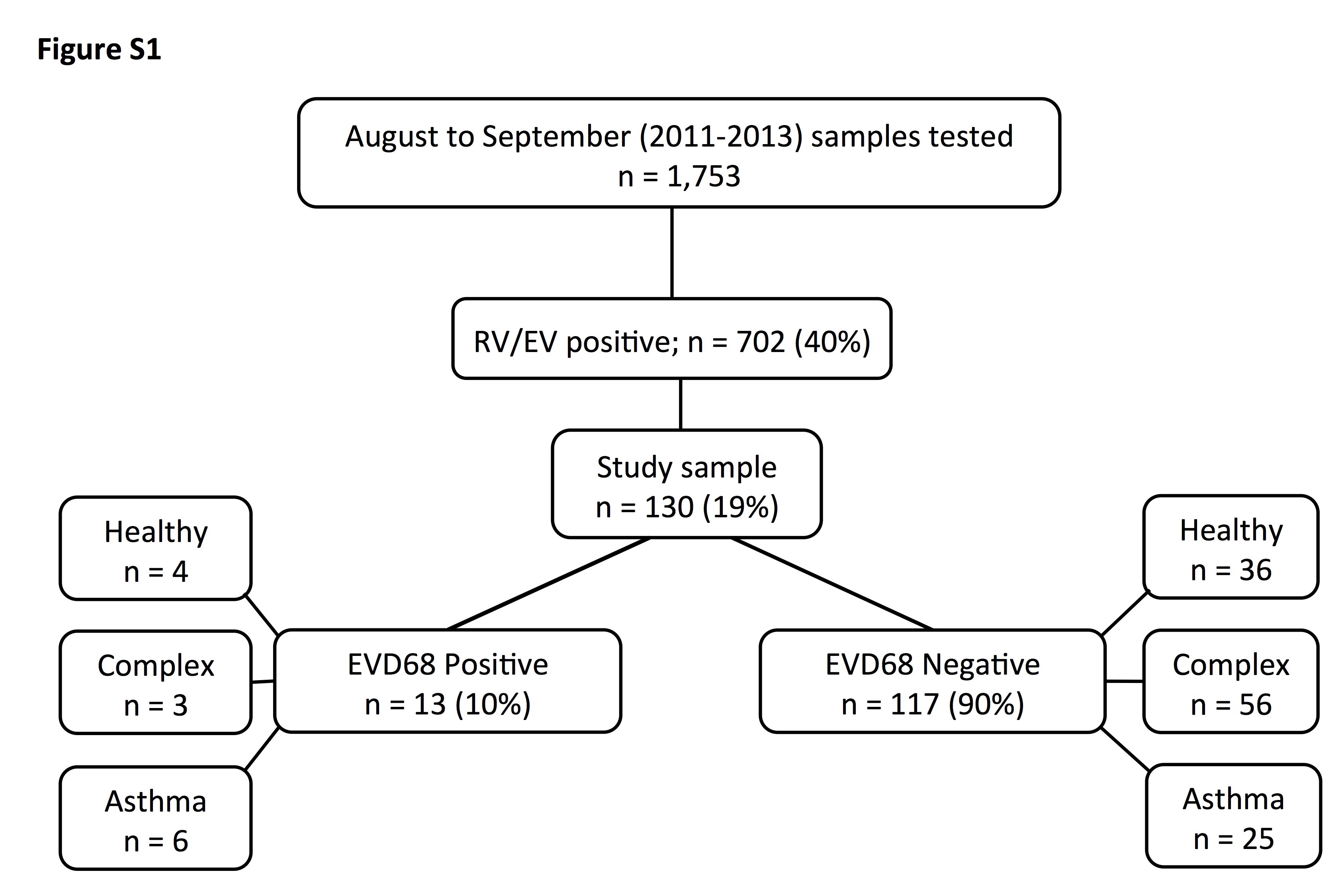

Supplement: S1 Fig — From August 12th to September 15th in 2011, 2012 and 2013 samples were selected randomly based on availability, integrity and amount of specimen. Samples included in the analyses were linked with patient characteristics. (TIFF) [file pone.0167111.s001.tiff]

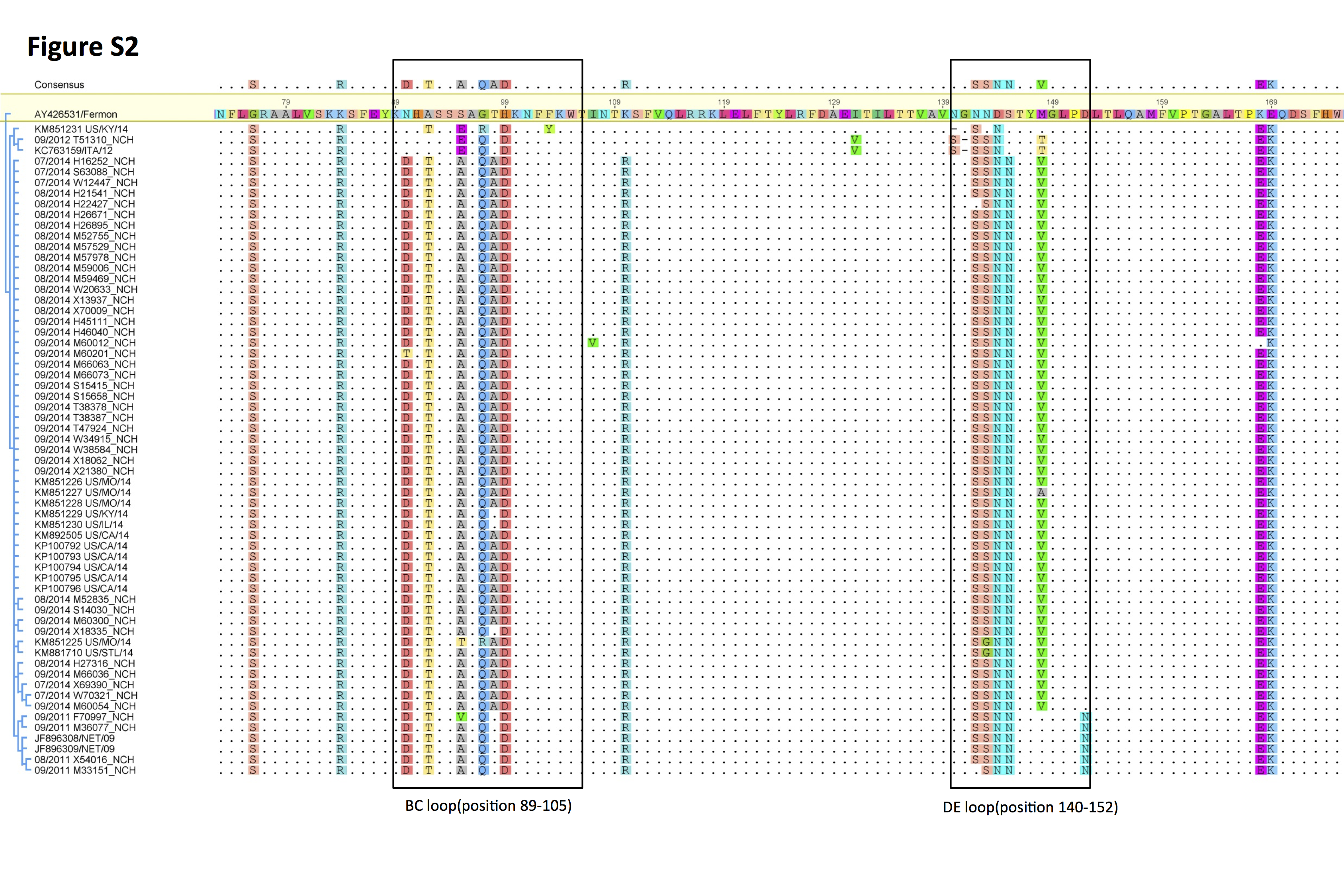

Supplement: S2 Fig — Partial VP1 protein sequences of NCH strains and selected strains from other areas were compared with the Fermon strain. Colored squares indicate the aminoacid differences compared to the Fermon strain while dots indicate amino acids identical to those in the Fermon strain. The BC loop and the DE loop are included in the black box. (TIFF) [file pone.0167111.s002.tiff]
